# Deciding a Treatment Plan for an Older Patient With Severe Idiopathic Pulmonary Fibrosis: A Case Report

**DOI:** 10.7759/cureus.34154

**Published:** 2023-01-24

**Authors:** Yusuke Akashi, Yuta Horinishi, Chiaki Sano, Ryuichi Ohta

**Affiliations:** 1 Family Medicine, Shimane University Medical School, Izumo, JPN; 2 Community Care, Unnan City Hospital, Unnan, JPN; 3 Community Medicine Management, Shimane University Faculty of Medicine, Izumo, JPN

**Keywords:** advanced care planning, idiopathic pulmonary fibrosis, interstitial pneumonia, rural hospital, general medicine

## Abstract

Idiopathic pulmonary fibrosis (IPF) is a group of diseases in which the main loci of lesions, mainly inflammatory and fibrotic, are within the interstitium of the alveolar and bronchiolar regions. Steroid therapy is the standard treatment for acute exacerbation of IPF, whereas antifibrotic agents are the standard treatment for chronic IPF. However, the vulnerability of older patients indicates that these treatments may be discontinued. Here, we report the case of an 86-year-old woman who had a dry cough for over a year and was subsequently diagnosed with IPF based on imaging studies. After using steroid pulses to treat acute exacerbations, the patient was transitioned to the chronic management phase, and time was allowed to plan the patient’s advanced care with her family. The use of high-dose steroids in older patients with frailty is contraindicated. This case emphasizes the importance of considering initial intensive treatment for IPF in older patients for better palliative care.

## Introduction

Idiopathic pulmonary fibrosis (IPF) is a group of diseases in which the main loci of lesions, mainly inflammatory and fibrotic, are within the interstitium of the alveolar and bronchiolar regions [1]. The pathogenesis of IPF is thought to involve decreased pulmonary diffusion capacity and compliance [2]. Clinical signs include chest symptoms such as dry cough, dyspnea on exertion, and possibly fever during the acute phase. Of the idiopathic interstitial pneumonia, IPF is the most prevalent (80-90%), followed by idiopathic nonspecific interstitial pneumonia (5-10%) and idiopathic organizing pneumonia (1-2%) [3]. In a survey of IPF in Japan, the incidence rate was 2.23 per 100,000 people, and the prevalence was 10 per 100,000 people. Moreover, most patients are asymptomatic in the early stages of IPF [1].

IPF diagnosis is based on the exclusion of interstitial pneumonia and other diseases with diffuse pulmonary shadows, such as collagen disease and drug-induced interstitial pneumonia of known etiology [4]. A definitive diagnosis should be based on the histopathological diagnosis after a surgical lung biopsy. Steroid therapy, including pulsed therapy, is recommended for acute exacerbation of IPF; however, in the chronic phase, treatment with steroids and immunosuppressive agents is not recommended, with antifibrotic agents, such as pirfenidone and nintedanib being the standard treatment choices for chronic IPF. Despite this, there is no established evidence for the intensive management of interstitial pneumonia in older patients. This case highlights the importance of considering intensive treatments for IPF in older patients and facilitating effective decision-making in palliative care.

## Case presentation

An 86-year-old woman presented to our hospital with a mild fever and dry cough that had persisted for over one year. One day before the visit, she went to the outpatient department of internal medicine of a rural community hospital with a fever of 37 °C and a worsened cough without any history of infection. On admission day, the dry cough worsened with dyspnea, so she came to the hospital's emergency department. The patient’s medical history included hypertension, gastric ulcers, atrophic gastritis, and constipation. Her prescribed medications included amlodipine, losartan potassium, clarithromycin, and carbocisteine.

On admission, her consciousness was alert. Her vital signs were as follows: body temperature, 38.1 °C; blood pressure, 116/63; pulse rate, 100 bpm; respiratory rate, 24 breaths/min; and SpO_2_, 92 (room air). Physical examination revealed fine bilateral crackles on the chest without any murmur on the heart. She had no abnormalities in her neck, skin, joints, or legs. Sputum Gram staining showed phagocytosis of gram-positive diplococcus and gram-negative rod-shaped bacteria. Her chest X-ray showed bilateral infiltration on the lower parts without any abnormality of an electrocardiogram. The patient was diagnosed with moderate community-acquired pneumonia and treated with sulbactam/ampicillin (SBT/ABPC) of 6 g per day. However, her respiratory condition gradually exacerbated on the second day of admission. She was diagnosed with exacerbated interstitial pneumonia triggered by bacterial pneumonia due to a sudden increase in oxygen demand, basal lung cellulite, traction bronchiectasis on chest computed tomography (CT), aggravation of frosted shadows in both lungs, KL6 level of 878, elevated lactate dehydrogenase (LDH) level of 294, and malnutrition from a low serum albumin level (Table 1).

**Table 1:** Initial laboratory data of the patient

Parameter	Level	Reference
White blood cells	18.5	3.5–9.1 × 10^3^/μL
Neutrophils	92.0	44.0–72.0%
Hemoglobin	10.8	11.3–15.2 g/dL
Platelets	27.8	13.0–36.9 × 10^4^/μL
Albumin	2.9	3.8–5.3 g/dL
Total bilirubin	1.3	0.2–1.2 mg/dL
Aspartate aminotransferase	22	8–38 IU/L
Alanine aminotransferase	12	4–43 IU/L
γ-Glutamyl transpeptidase	15	<48 IU/L
Lactate dehydrogenase	294	121–245 U/L
Uric acid	5.4	3.0–6.9 mg/dL
Blood urea nitrogen	21.7	8–20 mg/dL
Creatinine	0.77	0.40–1.10 mg/dL
Estimated glomerular filtration rate	53.1	>60.0 mL/min/L
Serum sodium	130	135–150 mEq/L
Serum potassium	3.4	3.5–5.3 mEq/L
Serum Chloride	6	98–110 mEq/L
Serum Calcium	8.5	8.8–10.2 mg/dL
Serum phosphorus	3.3	2.7–4.6 mg/dL
Serum magnesium	2.2	1.8–2.3 mg/dL
Serum glucose	147	70–110 mg/dL
hemoglobin a1c	5.9	5.0–6.2%
C-reactive protein	29.58	<0.30 mg/dL
Erythrocyte sedimentation rate	80	3–15 mm
KL-6	878	105.3–401.2 U/ml
Surfactant protein D	192	-110 ng/ml
Surfactant protein A	94.5	-43.8 ng/ml
Anti-citrullinated protein antibody	<0.6	<0.6 U/ml
Protease 3-anti-neutrophil cytoplasmic antibody	<1.0	-3.5 U/ml
Myeloperoxidase-Anti-neutrophil cytoplasmic antibody	<1.0	-3.5 U/ml
Arterial blood gas		
pH	7.457	7.35–7.45
PO_2_	56.3	75.0–100.0
PCO_2_	36.8	35.0–45.0
Base excess	2.3	-3.0 to 3.0
Lactate	0.8	0.5–1.6 mmol/L
Urine test		
Leukocyte	Negative	Negative
Nitrite	Negative	Negative
Protein	(1+)	Negative
Glucose	Negative	Negative
Urobilinogen	Negative	Negative
Bilirubin	Negative	Negative
Ketone	Negative	Negative
Blood	(1+)	Negative
Bacteria	Netative	Negative

**Figure 1:**
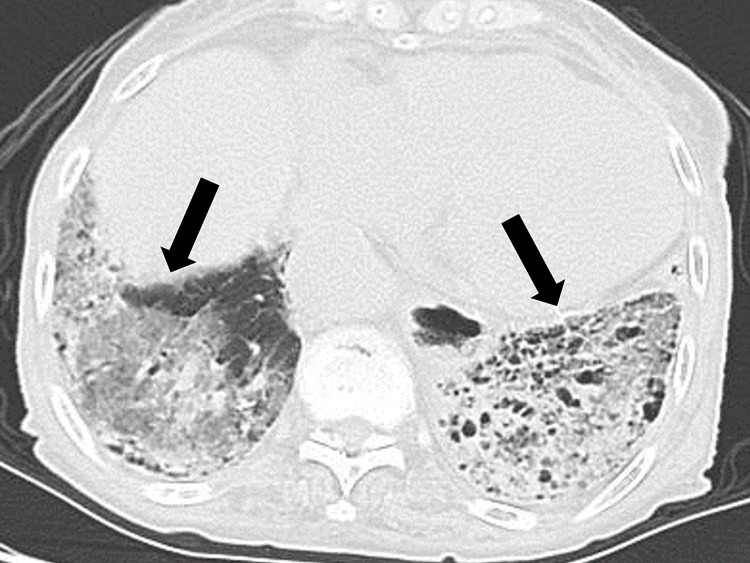
Computed tomography of the chest showing bilateral interstitial infiltration with honey-comb appearance (black arrows).

Based on chest CT features, the patient was diagnosed with IPF (Figure 1).

The patient was intubated, and ventilated to manage the rapidly increasing oxygen demand, marked tachypnea, and increased respiratory effort. Steroid pulse therapy (methylprednisolone (mPSL) 1000 mg/day for three days) was effective for the exacerbation of interstitial pneumonia, with the initial P/F of approximately 120 improving to 276, meaning the patient was extubated.

After the treatment of mPSL, the prednisolone dose started at 30 mg, and azathioprine was at 25 mg. A week later, the azathioprine was increased to 50 mg owing to worsening of the frosted shadows on chest CT, elevated LDH, surfactant protein-D of 192, surfactant protein-A of 94.5, and inflammatory findings. LDH began to fall, and the mPSL dose was tapered to 20 mg without worsening the respiratory status. The duration of transient alleviation allowed palliative care to be discussed with the patient and her family. During the discussion, the patient expressed willingness to accept intensive care treatment for her symptoms. Owing to the strong respiratory effort required while eating, the patient could not consume/obtain sufficient energy. Moreover, she was unable to undergo rehabilitation to improve her motor skill. A gastrostomy tube was placed to secure the nutritional route, and rehabilitation was performed while ensuring constant nutrition.

The patient developed acute hyponatremia, probably caused by tube feeding, and subsequently developed a consciousness disorder. At the same time, probably due to metabolic alkalosis, she developed hypokalemia; therefore, she was intubated and ventilated a second time. The patient hoped for intensive care; however, this was not effective. The patient’s family also agreed with her wishes, and the patient was treated for a second time with a ventilator and tracheal intubation.

The patient’s hyponatremia, hypokalemia, and consciousness subsequently improved, and spontaneous respiration recovered. However, spontaneous respiration was unstable due to carbon dioxide retention; therefore, a tracheostomy was performed. Several days after the tracheostomy, acute-onset tachypnea, decreased oxygenation, tachycardia, and hypotension were observed. Considering the patient's long-term management with ventilation and immunosuppressed state, the possibility of exacerbation of interstitial pneumonia due to complications such as ventilator-associated pneumonia, sepsis, and pneumocystis pneumonia was considered. The patient was unresponsive to treatment and died eight days later.

## Discussion

This case highlights the importance of an effective diagnostic process for IPF in older patients with frailty. Moreover, the initial intensive treatment of IPF exacerbation may enable discussion of palliative care with the family members of these patients.

The diagnosis of IPF in older patients should be based on multiple medical information sources without invasive procedures [4]. The patient's chest CT showed diffuse frosted shadows in both lungs and findings suspicious of cellulite lung with traction bronchiectasis at the base of the lower lobe of the left lung. There were no physical findings or laboratory data to suggest either infectious diseases or collagen diseases, such as rheumatoid arthritis, hypersensitivity pneumonitis, drug-induced pneumonia, or asbestos lung, and the cause of interstitial pneumonia was unclear; therefore, a diagnosis of IPF was made as the patient was in a secondary hospital, a surgical biopsy was not performed, and CT images showed a usual interstitial pneumonia (UIP) pattern [5]. In this case, the patient responded to a steroid pulse during an acute exacerbation; therefore, PSL at 0.5 mg/kg/day was tapered off, but the patient was exacerbated again. Although the combination of steroids and immunosuppressive drugs is not generally recommended in the chronic phase of IPF treatment, azathioprine was added because of the patient's high SP-D level and residual inflammatory findings [6]. The combination of steroids and azathioprine was effective, resulting in improved temporary oxygenation and exercise tolerance.

Initial intensive treatment of IPF in older patients can facilitate the discussion of advanced care planning and palliative care. We discuss treatment plans for idiopathic interstitial pneumonia with tube feeding in very old patients, including their families, after pulse treatment with methylprednisolone. Through this discussion, the patient and family can share their thoughts, allowing physicians to progress confidently with the patient’s treatment. Effective and efficient treatments for very old patients require advanced directives and palliative care based on their wishes [7,8]. Moreover, intensive treatment among older patients tends to be avoided because of various complications [9,10]. However, as in this case, intensive treatment at an initial stage can mitigate communication difficulties for older patients, enabling them to express their treatment wishes and improving treatment satisfaction for both the patient and their family. Treatment plan decision-making can be stressful for general physicians, as many older patients cannot express their treatment wishes owing to a lack of decision-making capacity [11]. In situations in which older patients have the potential to regain their decision-making abilities with treatment, general physicians should consider intensive treatment.

The treatment of IPF in older patients with immunosuppressants is controversial in palliative care. Current international treatment guidelines for idiopathic interstitial pneumonia do not recommend immunosuppressive agents for the chronic phase of IPF [12]. Moreover, the indication for intensive care in very old patients, such as in this case, is still under consideration. This patient had high surfactant protein-D levels and residual inflammatory findings; therefore, azathioprine was added during mPSL tapering after steroid pulse therapy. The combination of steroids and azathioprine was successful and improved temporary oxygenation and exercise tolerance. Older patients have a wide range of physiological variabilities and immunity [13,14]. Therefore, physicians cannot determine health conditions based only on age. The effective treatment of older patients with exacerbation of IPF requires comprehensive assessment by general physicians as system-specific specialists.

Acute hyponatremia associated with tube feeding may have contributed to impaired consciousness, which led to second tracheal intubation. The prevalence of moderate chronic hyponatremia ranges from 2% to 4% in the general population, 7% to 11% in older outpatients, and 42% in hospitalized patients [15]. Symptoms of hyponatremia, such as impaired consciousness and nausea, differ between acute and chronic stages, with cerebral edema being the main pathophysiology. There are two defense responses to cerebral edema in acute hyponatremia. The first is a hydrostatic pressure change from the brain to the cerebrospinal fluid, followed by a hydrostatic pressure change in systemic circulation [16]. The second is the depletion of ions in the brain cells, which reaches a new steady state after approximately three hours. There are limitations to both mechanisms, with symptoms such as disorientation, altered consciousness, and nausea, which can potentially occur with cerebral edema [17]. However, chronic hyponatremia has few symptoms, as the brain can compensate for hydrostatic pressure changes between the brain and cerebrospinal fluid and electrolyte concentrations within brain cells. Tube feeding may effectively reduce the risk of hyponatremia; however, the low sodium content of tube feedings, combined with the predisposition of older patients to the syndrome of inadequate antidiuretic hormone, makes them more prone to hyponatremia [17]. This case of acute hyponatremia was considered to have resulted in impaired consciousness.

## Conclusions

In the chronic phase of IPF treatment, inflammatory markers, such as surfactant protein-D and surfactant protein-A, are also used. A combination of steroids and immunosuppressive agents may be effective when a coexisting inflammatory pathology is presumed. Hyponatremia is a frequent cause of electrolyte abnormalities in the older population; however, acute progressive hyponatremia is likely to become symptomatic and requires early correction. In addition, this case suggests that the initial intensive treatment of IPF exacerbation may facilitate palliative care decisions involving older patients and their family members.
